# Impact of Cooking Processes on Volatile Flavor Compounds and Free Amino Acids in Fish Sauce

**DOI:** 10.3390/foods14040586

**Published:** 2025-02-10

**Authors:** Jin-Xiu Xu, Xin Zhang, Bai-Feng Fu, Xin-Yu Qiao, Zhen-Yu Wang, Xian-Bing Xu, Shu-Zhen Cheng, Ming Du

**Affiliations:** 1 Key Laboratory of Food Nutrition and Health of Liaoning Province, School of Food Science and Technology, Dalian Polytechnic University, Dalian 116034, China; jxiuxu@163.com (J.-X.X.); zhangxiniiii@163.com (X.Z.); fubaifeng121@163.com (B.-F.F.); qiaoxy98@163.com (X.-Y.Q.); wangzy0506@outlook.com (Z.-Y.W.); xianbingxu@dlpu.edu.cn (X.-B.X.); 2 SKL of Marine Food Processing & Safety Control, National Engineering Research Center of Seafood, School of Food Science and Technology, Dalian Polytechnic University, Dalian 116034, China

**Keywords:** volatile flavor compounds, free amino acids, different cooking processes, fish sauce

## Abstract

Fish sauce is a widely used condiment in cooking. However, the effects of various cooking processes on its quality remain poorly understood. This study evaluated the quality of fish sauce subjected to various cooking methods (boiling for 10, 30, and 60 min and stir-frying for 10, 30, and 60 s) using sensory evaluation, electronic nose, gas chromatography-mass spectrometry, and free amino acid analysis. Thermal cooking processes significantly altered the flavor profile of the fish sauce. A total of 38 volatile compounds were identified and quantified, 10 of which were screened as key aroma-active compounds based on their high odor activity values (OAVs ≥ 1). The total OAV values of key flavor compounds were lowest in unheated fish sauce (346.51) and varied from 347.64 to 707.40 in heated fish sauce. The maximum total free amino acid contents in boiled and stir-fried fish sauce were 4862.52 mg/100 mL and 4922.49 mg/100 mL, respectively, which were significantly higher than those in unheated fish sauce (4577.54 mg/100 mL) (*p* < 0.05). Pearson correlation analysis indicated that temperature modulates the production of key flavor compounds in fish sauce by influencing amino acid metabolism. These findings provide valuable insights into the application of fish sauce in food production and culinary practices.

## 1. Introduction

Fish sauce is a vital condiment that enhances the flavor and appeal of various foods, and it is widely popular in Southeast and East Asian countries [1,2]. Fish sauce is typically used in two ways: as a fresh, salty seasoning to flavor food, or as an ingredient during cooking or heating [3]. Cooking significantly alters the composition of free amino acids (FAAs), fatty acids, and carbohydrates [4,5]. These substances contribute to the flavor formation in fish sauce [6]. Therefore, cooking is believed to significantly influence the flavor of fish sauce. A better understanding of fish sauce flavor after cooking is crucial for improving the sensory quality of cooked food. Therefore, it is important to clarify the flavor characteristics of fish sauce under various cooking processes. However, little research has been conducted on the changes in fish sauce flavor quality after cooking.

Heating affects the flavor quality of food, including its aroma and taste [5]. Aroma is considered a key indicator of fish sauce quality and consumer acceptance [7]. Several physical and chemical changes occur during food heating, resulting in alterations in volatile compounds and the formation of new flavor compounds [8]. For example, benzaldehyde and phenylacetaldehyde, produced by the Maillard reaction of phenylalanine, are present in all heat-treated camellia seeds [9]. Thermal treatments also induce the production and release of volatile compounds in soy sauce [3], bamboo shoots [8], French fries [10], and duck meat [11]. These changes in volatile compounds contribute to a stronger and more abundant odor in the food. Additionally, high temperatures during cooking cause the loss of certain odorants.

FAAs, another key indicator of fish sauce quality, significantly influences its taste and overall flavor profile [12]. The content of FAAs is also influenced by cooking processes [13]. Proteolysis products formed during thermal processing may contribute to the accumulation of FAAs [4]. However, thermal processing simultaneously depletes FAAs, leading to Strecker degradation and the generation of various volatile compounds [9]. This study aimed to investigate the effects of different cooking processes (boiling and stir-frying) and time on volatile flavor compounds and FAAs in fish sauce, offering practical guidance for its use in food production and culinary practices. Sensory evaluation, electronic nose (E-nose), and gas chromatography-mass spectrometry (GC-MS) were used to analyze the overall flavor profile of fish sauce. The contribution of key flavor compounds to aroma was then studied, as well as the differences between unheated and cooked fish sauce (boiled and stir-fried). FAAs content was also used to evaluate the quality of fish sauce subjected to different cooking processes.

## 2. Materials and Methods

### 2.1. Materials and Chemicals

Fish sauce was produced by Shantou Fish-sauce Factory Co., Ltd., Shantou, China. The fish sauce, produced in 2021, adhered to the GB 10133-2014 standards (National health and family planning commission, 2014) [14] and contained marine fish, salt, water, caramel color, and sodium benzoate. Cyclohexanone was purchased from Aladdin Reagent (Shanghai) Co., Ltd., Shanghai, China. C7-C30 n-alkanes, obtained from Sigma Aldrich Ltd., St. Louis, Mo, USA. (Analytical Standard), were used to calculate retention indices (RI) [15].

### 2.2. Sample Preparation

Boiling and stir-frying methods commonly used in China were simulated with modifications, based on the approach of Wang et al. [3]. For boiling, 500 mL of unheated fish sauce (U) was placed in a 1 L Erlenmeyer flask, sealed with tin foil (volume assumed to be preserved), and heated in a constant-temperature oil bath (DF101S, Shanghai Lichenbangxi Instrument Science and Technology Co., Ltd., Shanghai, China) at 100 °C with magnetic stirring for 10, 30, and 60 min. The boiled fish sauce samples were named B10, B30, and B60, respectively. The boiling process was repeated twice, and samples with the same boiling time were mixed. For stir-frying, the pan was preheated to 220 °C using an induction cooker (WK2102, Midea Group Co., Ltd., Foshan, China) at 1600 W for approximately 1 min, as measured with an infrared thermometer (DECTMM520C, Delixi (Wuhu) Network Technology Ltd., Wuhu, China). Then, 200 mL of fish sauce was added to the pan for 10, 30, and 60 s to obtain samples F10, F30, and F60. This stir-frying process was repeated five times, and samples with the same stir-frying time were mixed. The remaining volumes of the stir-fried samples were 0.94%, 0.79%, and 0.62% for F10, F30, and F60, respectively. Ultrapure water and salt were added to adjust the volume and salinity, which was measured using a digital salinity meter (DSM-25, Shanghai Lichenbangxi Instrument Science and Technology Co., Ltd., Shanghai, China) to a consistent level. All samples were stored at −20 °C until analysis.

### 2.3. Sensory Evaluation

A sensory panel of 9 members (4 men and 5 women, aged 20–30) from the Protein Science and Technology Research Group at Dalian Polytechnic University evaluated the flavor of fish sauce using quantitative descriptive analysis (QDA). Ten sensory descriptors related to fish sauce flavor were selected from literature and panelists’ descriptions. Participants rated the flavor attributes of each sample on a scale from 1 (unperceived) to 10 (extremely strong).

### 2.4. E-Nose Analysis

An E-nose (PEN3.0, Airsense Analytics GmbH, Schwerin, Germany) equipped with 10 metal oxide sensors, was used to analyze the volatile flavor profile of fish sauce subjected to different cooking processes. The sensors were cleaned with air for 200 s before measurement. For each sample, 0.50 g was placed in a 20 mL glass vial, which was sealed with a Polytetrafluoroethylene (PTFE)-coated cap. The E-nose probes were inserted into the sealed vial for a 100 s measurement. Sensor cleaning took 100 s for the same sample and 200 s for different samples. Data was processed using winmuster E-nose software (version 1.6.2).

### 2.5. HS-SPME GC-MS Analysis

Headspace solid-phase microextraction (HS-SPME) was employed to extract volatile compounds from fish sauce samples. Briefly, 5 g of each fish sauce sample, 2 g of sodium chloride, and 5 μL of cyclohexanone were transferred to a 20 mL headspace vial. The vial was sealed with a PTFE silicone cap and shaken to ensure thorough mixing. A dynamic equilibrium between absorption and release of volatile compounds was established by heating the samples in a magnetic stirrer (DF101S, Yuhua Instrument Co., Ltd., Shanghai, China) at 60 °C for 20 min. The DVB/CAR/PDMS (Supelco Analytical, Bellefonte, PA, USA) extraction head was inserted for 40 min, followed by desorption at 250 °C in the GC/MS (7890B-5977A, Agilent Science and Technology (China) Co., Ltd., Beijing, China) injector port for 10 min.

Volatile compounds from the fish sauce samples were analyzed using GC-MS. The GC oven was initially set to 50 °C for 3 min, then increased to 170 °C (hold for 3 min) at a rate of 3 °C/min, and finally to 230 °C (hold for 5 min) at 4 °C/min. The temperatures of the MS transfer line and ion source were set to 280 °C and 230 °C, respectively. The ion source was FID, and MS detection was performed over the range of 35–500 *m*/*z*. Volatile compounds were identified based on RI and the mass spectrometry databases NIST 14. Semi-quantification of fish sauce was performed by dividing the specific GC peak area of each volatile by the cyclohexanone peak area, then multiplying the result by the cyclohexanone quantity [16].

### 2.6. OAVs Calculation

OAVs were calculated as the ratio of the concentration of each odorant (C) to its respective threshold (OT) in water and were used to evaluate their contribution to the overall volatile flavor profile [7]. Compounds with OAV ≥ 1 are considered to significantly contribute to the aroma, with higher OAV indicating a greater contribution [17].

### 2.7. FAAs Analysis

Following the method of Zhang et al. [18], 5 mL of each fish sauce sample was mixed with 15 mL of acetone and centrifuged at 10,000× *g* for 10 min. The supernatant was evaporated, reconstituted in 0.02 M HCl, and filtered through micropore filters with a pore size of 0.22 μm. The resulting samples were analyzed using an automatic amino acid analyzer (LA8080, Hitachi Ltd., Tokyo, Japan).

### 2.8. Statistical Analysis

All measurements were performed in triplicate for each sample. A one-way analysis of variance (ANOVA) was conducted to evaluate statistical differences (*p* < 0.05). Radar plots and stacked graphs were generated using Origin 2024. A heatmap of flavor compounds was created using TBtools software (v1.0987671). Pearson correlation analysis (PCA) was performed using Origin 2024.

## 3. Results and Discussion

### 3.1. Sensory Characterization

**Figure 1:**
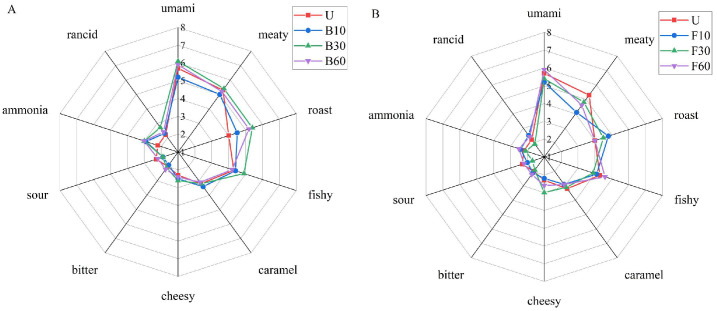
Radar plot based on sensory score of fish sauce subjected to boiling (**A**) and stir-frying (**B**). U (unheated); B10, B30, and B60 (heated at 100 °C for 10, 30, and 60 min, respectively); F10, F30, and F60 (heated at 220 °C for 10, 30, and 60 s, respectively).

As shown in Figure 1, fish sauce exhibits prominent sensory properties, including umami, meaty, fishy, roast, and caramel flavors, all of which received high ratings across all samples, consistent with previous studies [19,20]. Boiling significantly increased the roasting aroma and ammonia in all samples, likely due to the formation of trimethylamine and pyrazine. Moreover, the fishy taste intensified after 30 min of boiling. The umami taste diminished after 10 min, possibly due to changes in FAAs. During stir-frying, flavor changes in fish sauce primarily manifest in the meat and roast profiles, with the meat flavor diminishing after stir-frying for over 10 min. Previous studies have shown that heterocyclic compounds and their derivatives contribute to the meaty flavor of foods [21]. The roast flavor of fish sauce intensifies after stir-frying for 10 and 30 s but diminishes after 60 s. Overall, cooking methods and durations significantly affect the aroma profile of fish sauce.

### 3.2. E-Nose Evaluation of Flavor Profile

**Figure 2:**
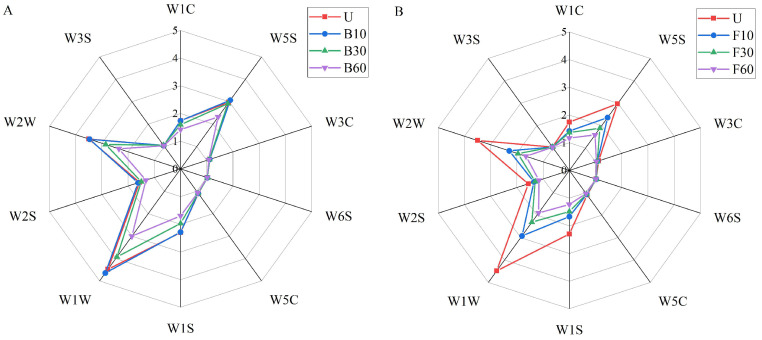
Radar plot based on Electronic-nose (E-nose) data and representing the overall aroma profiles of fish sauce subjected to boiling (**A**) and stir-frying (**B**). U (unheated); B10, B30, and B60 (heated at 100 °C for 10, 30, and 60 min, respectively); F10, F30, and F60 (heated at 220 °C for 10, 30, and 60 s, respectively).

The E-nose was employed to evaluate the overall flavor profile of fish sauce samples exposed to various cooking processes (Figure 2). The E-nose radar plot revealed that the response characteristics of the 10 sensors varied among the samples. Sensors W1C, W3C, W6S, W5C, W2S, and W3S exhibited lower signal intensities across all samples, suggesting that aromatic components, including hydrogen, arom-aliph, broad-alcohol, and methane-aliph, are present at low concentrations in the fish sauce flavor. Fish sauce samples exhibited stronger responses to W5S, W1S, W1W, and W2W, corresponding to higher concentrations of broad-range compounds, broad-methane, and sulfur-organic compounds. Additionally, variations in sensor response allowed the differentiation of fish sauce samples according to cooking time. The B10 group could not be distinguished from the U group, indicating that boiling for 10 min had a minimal impact on the overall aroma profile. The other groups were distinct from the unheated group, with longer heating times resulting in greater separation along the sensor axis, especially for stir-fried samples. Sensor response intensities on radar plots demonstrated that both cooking methods and cooking times significantly affect the fish sauce flavor profile. Moreover, cooking methods have a more pronounced impact on the aroma of fish sauce. Thus, a molecular sensory science approach was employed to identify changes in the flavor of fish sauce after cooking.

### 3.3. Qualitative and Semi-Quantification Analysis of Volatile Compounds in the Fish Sauces

**Figure 3:**
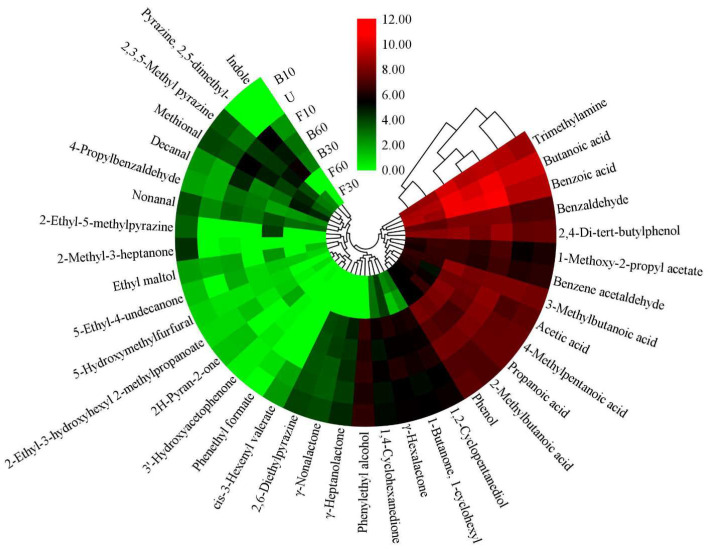
Heatmap visualization of volatile compounds in fish sauces following different cooking processes. U (unheated); B10, B30, and B60 (heated at 100 °C for 10, 30, and 60 min, respectively); F10, F30, and F60 (heated at 220 °C for 10, 30, and 60 s, respectively).

**Figure 4:**
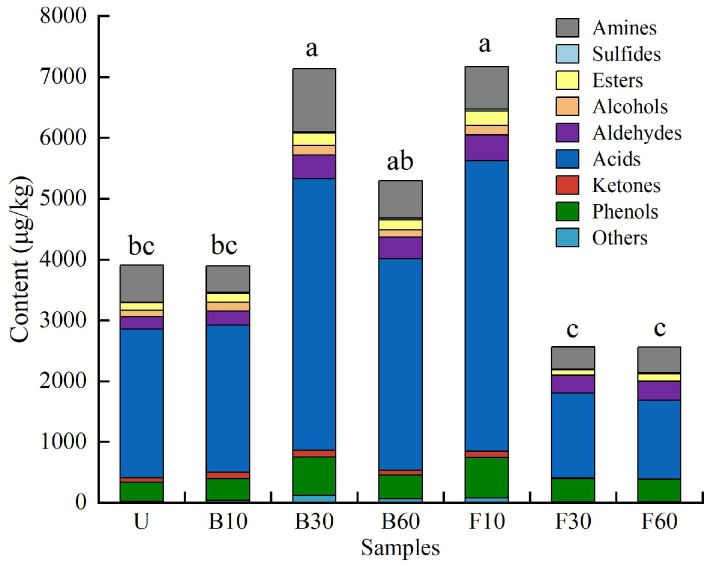
Stacked graph of volatile compounds in fish sauces following different cooking processes. U (unheated); B10, B30, and B60 (heated at 100 °C for 10, 30, and 60 min, respectively); F10, F30, and F60 (heated at 220 °C for 10, 30, and 60 s, respectively). Significant differences among different cooking fish sauces are indicated by different lowercase letters (*p* < 0.05).

The relative quantification of flavor compounds in fish sauce samples subjected to different cooking processes is shown as a heatmap (Figure 3). A total of 38 volatile compounds were detected across all fish sauce samples, including 1 amine, 1 sulfide, 7 esters, 2 alcohols, 5 aldehydes, 7 acids, 6 ketones, 3 phenols, and 6 others. The compounds identified by mass spectrometry (MS) were positively and negatively matched with a degree > 700. In addition, 32 compounds were confirmed by RI, with a deviation of less than 30 [22]. Figure 3 shows the identification of 33, 35, 35, 32, 34, 27, and 24 odorants in the U, B10, B30, B60, F10, F30, and F60 samples, respectively. The number of volatile compounds initially increased and then decreased with extended cooking times. The total volatile compound content (Figure 4) also reflects the effect of cooking processes on the flavor of fish sauce. The total volatile compound concentration in B30 was significantly higher than in the control group. Minimal variation was observed in total volatile compound concentration between U and B60, while no significant difference was observed between B10 and U. In the stir-fried samples, the intensity of volatiles in F10 increased significantly, while the intensities in F30 and F60 decreased slightly, remaining consistent with their respective volatile compound numbers. These results indicate that fish sauce exhibited higher concentrations of volatile compounds at moderate cooking times. This may occur because heating triggers the Maillard reaction, which generates flavor compounds. However, prolonged heating leads to the degradation of these compounds. Stir-frying involves higher temperatures, which accelerate the Maillard reaction, driving it more rapidly toward its final stage. Hierarchical clustering analysis (HCA) clustered the 38 volatile compounds and distinguished the fish sauce samples (Figure 3). The samples were divided into three clusters: F30 and F60, U, and B10, and F10, B30, and B60. This indicates that F30 and F60 shared similar aroma profiles, U resembled B10, and F10, B30, and B60 were like one another. The results of this study show that hot cooking significantly changes the flavor characteristics of fish sauce.

#### 3.3.1. Amine and Sulfide

The changes in flavor compounds detected in the fish sauce samples are presented in Figure 3. Trimethylamine is a key compound responsible for the characteristic fishy odor of aquatic products, distinguishing them from other foods. It is produced through the degradation of trimethylamine oxide by facultative anaerobic bacteria during continuous fermentation [23]. In this study, the concentration of trimethylamine ranged from 365.35 to 1038.54 μg/kg, with the highest concentrations in B30, followed by F10 and B60. These three groups also exhibited the highest concentrations of total volatile compounds (Figure 4). Methional is produced via the Strecker degradation of methionine, which serves as the precursor and is crucial in food flavor [24]. The methional content was significantly affected by cooking processes. Excluding F30, the methional content was higher in cooked samples than in unheated samples in this study.

#### 3.3.2. Esters and Alcohols

Carboxylic esters are formed through the esterification of alcohols and acids, while lactones are produced by the cyclization of hydroxycarboxylic acids [23]. Esters generally impart floral and fruity notes (short-chain esters) or fatty flavors (long-chain esters), while γ-lactones are sweet compounds that enhance aroma. Seven esters, including three lactones, were identified and quantified in the fish sauce samples. As cooking temperature and time increase, the number of esters in fish sauce decreases, likely due to the decomposition of heat-sensitive esters. However, the levels of heat-resistant esters increased during cooking. Alcohols are produced through lipid oxidation, carbohydrate metabolism, and amino acid decarboxylation [25]. As shown in Figure 3, phenylethyl alcohol had the highest concentration in B30, while trans-1,2-cyclopentanediol was most abundant in F10, followed by B30. The alcohol concentration in boiled samples was higher than in unheated samples. However, alcohol content decreased after stir-frying, except for 1,2-cyclopentanediol and phenylethyl alcohol in F10. Therefore, the cooking process should be optimized to enhance the flavor of fish sauce.

#### 3.3.3. Aldehydes

Previous studies have shown that aldehydes primarily arise from lipid oxidation or are produced through Maillard-induced amino acid degradation and the interaction between lipids and the Maillard reaction [16]. Benzaldehyde imparts almond, nutty, and fruity odors [26]. Nonanal imparts rose, fresh, and fatty flavors [16]. Benzene acetaldehyde imparts a rosy-like aroma [27], while decanal contributes to a pleasant fatty, floral, and green aroma [10]. In this study, the concentrations of benzaldehyde, benzene acetaldehyde, nonanal, decanal, and 4-propylbenzaldehyde were highest in F10, F10, B10, F60, and F10, respectively, with values 2.13, 1.58, 3.09, 13.24, and 3.32 times higher than those in U. Furthermore, the aldehyde content in all other cooked samples was higher than in sample U, except for benzene acetaldehyde in F30. This suggests that the cooking process resulted in changes in aldehyde levels, thereby enhancing the flavor of fish sauce.

#### 3.3.4. Acids

Seven volatile organic acids were identified: acetic acid (pungent sour), propanoic acid (cheesy), butanoic acid (rancid), 3-methylbutanoic acid (cheese), 2-methylbutanoic acid (sweaty), 4-methylpentanoic acid (cheesy), and benzoic acid (faint, balsamic, urine) [3,16]. Most of these compounds exhibited unpleasant odors, and their total content was the highest among all fish sauce samples. In samples B30 and F10, the acid concentrations were 4467.41 μg/kg and 4777.82 μg/kg, respectively, while F60 had the lowest concentration at 1298.06 μg/kg (Figure 4). However, these acids generally contribute minimally to the overall flavor because of their high threshold values [1]. Moreover, some short-chain (C < 6) acids are thought to significantly influence the final aroma of fish sauce due to their low threshold values and strong odors [25].

#### 3.3.5. Ketones and Phenols

Ketones, which are formed similarly to aldehydes, are primarily produced through the breakdown of amino acids and the oxidation of unsaturated fatty acids [1]. In boiled samples, ketone content increased initially and decreased with prolonged processing, reaching a peak in B10 or B30. In contrast, stir-fried samples showed the highest ketone concentration at F10, followed by a decrease. Phenols, strongly associated with fruit flavors, are derived from aromatic amino acids such as tyrosine and phenylalanine [7,17]. The variation in the content of the three phenols identified in this study follows the trend observed for ketones. All phenols were affected by the cooking process, with 2,4-Di-tert-butylphenol showing the most pronounced changes. Analysis revealed that its concentration increased by 1.21-fold in B30 and 1.41-fold in F10 relative to U.

#### 3.3.6. Others

Pyrazines, commonly found in fermented foods, contribute to the grill aroma of foods [28]. Pyrazine, a key flavor compound, is frequently used as an indicator of food sensory quality. 2,6-Diethylpyrazine and 2,3,5-methylpyrazine occur in both unheated and certain cooked fish sauce samples. These compounds are formed via slow Maillard reactions during prolonged fermentation [3,23]. 2-Ethyl-5-methylpyrazine and 2,5-dimethylpyrazine were detected exclusively in cooked fish sauce, likely generated via Maillard reactions triggered by cooking. 5-Hydroxymethylfurfural is an intermediate product of the Maillard reaction [29]. Boiling accelerates the formation of 5-hydroxymethylfurfural, while stir-frying facilitates its involvement in subsequent reactions, leading to significant consumption.. Indole was identified only in B30, B60, and F10, contributing an off-flavor (animal and fecal odor) to fish sauce [11]. A previous study suggests that indole may form in food under specific cooking conditions [11].

### 3.4. Key Flavor Compounds Identified by OAV Analysis in the Fish Sauces

To further evaluate the contribution of each flavor compound to the overall aroma profile of fish sauce, OAVs, which account for both concentration and odor threshold, were calculated. Ten volatile compounds were screened as key aroma-active compounds based on their high odor activity values (OAV ≥ 1) (Table 1). Trimethylamine exhibited the highest average OAV, followed by decanal, methional, 2,3,5-methyl pyrazine, benzene acetaldehyde, 2-ethyl-5-methylpyrazine, nonanal, 2,6-diethylpyrazine, phenylethyl alcohol, and γ-nonalactone. The total OAVs of key odorants increased in all samples following heating. These findings differ from the total concentration of volatile substances, likely due to the high acid content in fish sauce, which significantly impacts the total volatile content. However, total OAVs are primarily driven by compounds with low odor threshold values. The observed changes in total OAVs align with a previous study, which reported increased total odor intensity scores, quantification, and OAV values in soy sauce after cooking [3].

The enhancement of food flavor during heat processing mainly depends on the Maillard reaction, which is critical for improving food acceptability [30]. This reaction involves pyrazines and aldehydes as key flavor compounds [30]. During the Maillard reaction, α-dicarbonyl compounds from sugars or α,β-unsaturated aldehydes from lipids react with amino acids to form structure-related aldehydes and α-amino ketones, which serve as precursors of pyrazines, an N-containing heterocyclic aromatic compound [31]. In boiled fish sauce, the concentration of methional increased with extended heating, while the levels of other aldehydes and pyrazines initially increased and then decreased. In stir-fried fish sauce, excluding nonanal and 2,6-diethylpyrazine, the concentrations of other aldehydes and pyrazines first increased, then decreased, and later increased again. The OAV values of seven key flavor aldehydes and pyrazines did not increase consistently with heating time and temperature. This may be because aldehydes are key intermediates in the Maillard reaction, which contribute to melanoid formation [32]. Pyrazine formation competes with melanoid formation [32]. In stir-fried fish sauce, these Maillard products are highly volatile and evaporate with water vapor [31]. Phenylethyl alcohol, with phenylacetaldehyde as its precursor, is also influenced by heating temperature during its formation and degradation [33]. During black tea drying, the concentration of phenylethyl alcohol is lower with sun drying (20–34 °C) than with hot-air drying (110–120 °C), but higher than with pan-fired drying (180–250 °C) [34]. These results are consistent with our findings. Moderate heating increases the OAV of phenylethyl alcohol, while excessive heating causes its degradation. The precursor of γ-nonalactone is 4-hydroxy acids, and heat drives their conversion into γ-nonalactone [35]. During heat treatment, ATP-related compounds are converted into nucleotides, which are then thermally degraded to trimethylamine [36,37]. A previous study has shown that trimethylamine content increases with heat treatment [37]. Heat may also cause a decrease in trimethylamine levels [38]. In this study, an increase in the OAV value of trimethylamine was observed in B30, B60, and F10, while it decreased in other samples. This may be because the degradation rate of trimethylamine in B10 is higher than its synthesis rate, while the highly volatile trimethylamine in F30 and F60 is lost during evaporation.

**Table 1:** Key volatile compounds in fish sauces following different cooking processes.

Volatile Compounds	Odor Threshold (μg/kg)	OAVs							Odor Description
		U	B10	B30	B60	F10	F30	F60	
Trimethylamine	2.4	251.75	182.94	432.73	257.00	288.73	152.23	174.00	Fishy
γ-Nonalactone	9.7	0.99	1.36	1.55	1.66	1.05	ND	ND	Sweet, creamy, coconut-like
Phenylethyl alcohol	45	1.40	1.95	2.14	1.53	1.92	ND	ND	Honey, rose
Benzene acetaldehyde	4	17.36	20.07	23.82	17.78	27.43	14.81	22.84	Rosy-like
Nonanal	1	4.25	13.15	12.17	6.50	9.63	8.22	7.43	Rose, fresh, fatty
Decanal	0.1	22.20	38.90	69.80	49.40	235.50	227.30	293.90	Fatty, floral, and green
Methional	0.4	30.98	36.05	37.35	68.35	97.85	24.25	57.40	Cooked potato
2-Ethyl-5-methylpyrazine	0.4	ND	23.40	40.60	ND	ND	ND	ND	Potatoes, roasted nuts
2,6-Diethylpyrazine	6	2.02	2.59	2.21	1.93	1.81	ND	ND	roasted, nutty, sweet
2,3,5-Methyl pyrazine	0.4	15.58	27.23	75.55	46.48	43.48	14.25	31.00	Nuts, chocolate, coffee
Total		346.51	347.64	697.91	450.63	707.40	441.06	586.57	

ND: Not detectedND. OAVs: Odor activity values. Odor threshold in water and odor description found in the literature and liber [10,16,17,23,27,39,40,41,42,43].

### 3.5. FAAs of Fish Sauces

Seventeen FAAs were identified in all fish sauce samples, and they are considered to significantly contribute to the flavor of the fish sauce (Table 2). As shown in Table 2, as heating time increased, the total FAAs content in the boiled sample initially increased, then decreased, but overall showed a significant increase. This suggests that heating promotes protein hydrolysis in fish sauce, resulting in the release of more amino acids. These findings agree with previous research on freshwater crayfish [40]. In stir-fried fish sauce, the total FAAs content initially decreases, then increases, and finally decreases again. This may result from the increased heating temperature, which promotes oxidative damage, amino acid side chain modification, and aromatic amino acid modification, leading to greater FAAs loss [44]. Consequently, the total amino acids content in F10 decreases significantly over a short period. However, the abundant protein in the fish sauce aggregates and gels at high temperatures, forming a three-dimensional protein network on the pan surface [45]. This protects the FAAs in the fish sauce, leading to a significant increase in total FAAs content in F30. However, this process hinders the formation of volatile flavor compounds, leading to the lowest concentration of key flavor compounds, such as trimethylamine, in F30. As heating time increased, the temperature of the fish sauce rose, resulting in a significant decrease in FAAs content. Additionally, due to the varying heat resistance of amino acids, after 60 min of cooking, the levels of umami and sweet amino acids decreased significantly compared to unheated fish sauce, whereas the levels of bitter and bittersweet amino acids increased.

### 3.6. Correlation Network Analysis of Key Volatile Compounds and FAAs of Fish Sauce Samples

**Figure 5:**
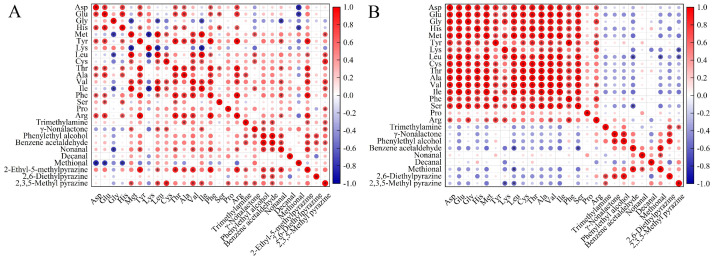
Pearson correlation analysis between key volatile compounds and free amino acids during boiling (**A**) and stir-frying (**B**). Symbol “*” represents *p* < 0.05.

This study demonstrated that the intensity of key aroma compounds and the content of FAAs in fish sauce varied with both cooking time and method. The degradation of FAAs was closely linked to the formation and content of key flavor compounds. The correlation between key aroma compounds and FAAs was analyzed to gain deeper insights into flavor formation during fish sauce cooking. As shown in Figure 5, during cooking, the content of FAAs in fish sauce (except for gly, lys, and his) was positively correlated with several flavor compounds. Some of these correlations were significant, the most notable being the positive correlation with pyrazine content. This may be due to the α-aminoketone produced in the amino acid-based Maillard reaction, which is the main precursor of pyrazine [31]. These findings are consistent with those reported by Zhou et al. [47], where key flavor compounds in grass carp were positively correlated with differential lipids under different freeze-thaw cycles and subsequent heat treatments. In stir-fried fish sauce, FAAs were primarily negatively correlated with key flavor compounds, although only a few correlations were statistically significant. This is consistent with the negative correlation between key flavor components and flavor precursors observed during the cooking of raw crayfish, as reported by Li et al. [40]. This suggests that cooking temperature, rather than cooking time, has a more significant effect on FAAs and key flavor compounds, as both proteolysis and the Maillard reaction are directly influenced by temperature [4].

## 4. Conclusions

The dynamic changes in volatile flavor compounds and free amino acids (FAAs) in fish sauce during cooking were evaluated in the present study. The diversity and abundance of volatile compounds in all cooked fish sauce samples (B10, B30, B60, F10, F30, and F60) were affected. High temperatures during fish sauce cooking result in the consumption, production, and release of volatile components, which enhance the intensity and complexity of the aroma. Ten compounds were identified as key flavor compounds. Cooking temperature, rather than cooking time, has a greater impact on FAAs and key flavor compounds. The optimal thermal processing condition for fish sauce was found to be boiling for 30 min, based on the results for volatile flavor compounds and FAAs. Carefully selecting the cooking time and method is essential for maintaining the quality of fish sauce. Stir-frying should generally be avoided, and cooking time should be carefully optimized. These findings offer theoretical guidance for developing cooking and processing conditions for dishes and food products containing fish sauce. However, this study has certain limitations. Different raw materials should be combined with fish sauce during heating to assess the applicability of the current findings in food production and culinary applications.

## Figures and Tables

**Table 2:** Free amino acids (FAAs) concentration (mg/100 mL) in fish sauces following different cooking processes.

Amino Acids	Average Content ± SD						
	U	B10	B30	B60	F10	F30	F60
Asp	3.81 ± 0.13 ^bc^	3.75 ± 0.04 ^bcd^	3.9 ± 0.07 ^b^	3.5 ± 0.18 ^cd^	3.91 ± 0.15 ^b^	4.72 ± 0.13 ^a^	3.42 ± 0.21 ^d^
Glu	43.8 ± 0.15 ^bc^	43.05 ± 0.25 ^cd^	44.33 ± 0.18 ^b^	39.63 ± 0.87 ^e^	43.46 ± 0.75 ^bcd^	53.31 ± 0.21 ^a^	42.46 ± 0.34 ^d^
Gly	25.77 ± 0.07 ^b^	22.33 ± 1.65 ^d^	23.5 ± 0.26 ^cd^	22.75 ± 0.49 ^d^	25.01 ± 0.29 ^bc^	28.7 ± 0.58 ^a^	22.84 ± 0.2 ^d^
His	42.71 ± 1.25 ^b^	42.11 ± 0.93 ^b^	42.95 ± 1.45 ^b^	38.32 ± 2.92 ^c^	45.8 ± 1.39 ^b^	51.51 ± 1.09 ^a^	44.05 ± 0.88 ^b^
Met	349.72 ± 0.75 ^d^	365.12 ± 0.8 ^c^	371.56 ± 0.91 ^b^	369.69 ± 2.52 ^b^	340.06 ± 1.21 ^e^	382.61 ± 0.71 ^a^	335.41 ± 2.94 ^f^
Tyr	176.82 ± 0.67 ^d^	184.06 ± 0.88 ^c^	190.55 ± 0.49 ^a^	172.15 ± 1.43 ^e^	185.13 ± 1.09 ^bc^	187.08 ± 0.92 ^b^	170.66 ± 1.16 ^e^
Lys	38.31 ± 1.41 ^a^	33.2 ± 2.99 ^b^	31.89 ± 0.27 ^b^	30.12 ± 0.33 ^b^	33.26 ± 0.71 ^b^	38.92 ± 1.12 ^a^	31.4 ± 0.2 ^b^
Leu	1489.3 ± 1.55 ^d^	1551.16 ± 0.27 ^c^	1583.84 ± 0.67 ^a^	1566.01 ± 9.15 ^b^	1440.22 ± 1.66 ^e^	1588.02 ± 3.49 ^a^	1444.08 ± 11.53 ^e^
Cys	6.25 ± 0.21 ^b^	6.67 ± 0.23 ^b^	6.91 ± 0.07 ^ab^	6.77 ± 0.62 ^b^	6.46 ± 0.24 ^b^	7.54 ± 0.12 ^a^	6.16 ± 0.17 ^b^
Thr	90.2 ± 0.18 ^cd^	92.3 ± 1.45 ^c^	97.56 ± 2.39 ^b^	88.28 ± 0.71 ^d^	96.96 ± 1.13 ^b^	111.11 ± 0.53 ^a^	90.88 ± 0.82 ^cd^
Ala	163.98 ± 1.34 ^c^	163.12 ± 0.25 ^cd^	167.37 ± 1.43 ^b^	161.18 ± 1.43 ^de^	165.65 ± 0.46 ^bc^	191.02 ± 0.73 ^a^	159.95 ± 1.29 ^e^
Val	399.26 ± 0.94 ^d^	409.88 ± 0.85 ^c^	419.52 ± 1.46 ^b^	406.06 ± 3.03 ^c^	399.84 ± 1.23 ^d^	437.81 ± 0.27 ^a^	387.82 ± 4.59 ^e^
Ile	708.12 ± 1.53 ^e^	738.29 ± 1.98 ^d^	755.31 ± 1.39 ^b^	749.28 ± 4.93 ^c^	692.37 ± 0.64 ^f^	768.38 ± 1.75 ^a^	692.7 ± 3.03 ^f^
Phe	882.33 ± 5.91 ^d^	921.82 ± 1.27 ^b^	951.3 ± 1.1 ^a^	875.85 ± 6.96 ^d^	900.7 ± 2.38 ^c^	906.77 ± 1.45 ^c^	863.26 ± 5.36 ^e^
Ser	16.85 ± 0.76 ^b^	17 ± 0.76 ^b^	16.86 ± 1.26 ^b^	15.72 ± 0.12 ^b^	16.68 ± 0.67 ^b^	19.31 ± 0.27 ^a^	16.12 ± 0.3 ^b^
Pro	29.71 ± 1.4 ^a^	30.93 ± 3.16 ^a^	34.03 ± 2.37 ^a^	31.04 ± 3.67 ^a^	33.49 ± 9.07 ^a^	34.73 ± 3.96 ^a^	30.23 ± 5.53 ^a^
Arg	110.59 ± 1.06 ^c^	115.39 ± 0.48 ^b^	121.14 ± 0.62 ^a^	105.77 ± 0.76 ^d^	110.25 ± 0.49 ^c^	110.95 ± 1.22 ^c^	107.39 ± 0.28 ^d^
Umami amino acids	47.61 ± 0.08 ^bc^	46.8 ± 0.27 ^cd^	48.23 ± 0.17 ^b^	43.13 ± 0.8 ^e^	47.37 ± 0.88 ^bc^	58.03 ± 0.31 ^a^	45.88 ± 0.32 ^d^
Sweet amino acids	296.8 ± 2.13 ^c^	294.75 ± 0.72 ^cd^	305.29 ± 3.88 ^b^	287.94 ± 2.68 ^e^	304.29 ± 2.39 ^b^	350.14 ± 0.41 ^a^	289.79 ± 2.1 ^de^
Bitter amino acids	3299.28 ± 7.7 ^d^	3437.43 ± 4.6 ^b^	3523.94 ± 2.02 ^a^	3401.62 ± 22.46 ^c^	3264.22 ± 6.76 ^e^	3501.76 ± 4.46 ^a^	3214.75 ± 21.02 ^f^
Bittersweet amino acids	927.6 ± 2.68 ^d^	954.52 ± 2.56 ^c^	978.14 ± 1.28 ^b^	942.68 ± 6.79 ^c^	916.89 ± 9.24 ^d^	1005.02 ± 6.96 ^a^	892.24 ± 9.41 ^e^
Total 17 AA	4577.54 ± 6.2 ^e^	4740.17 ± 7.81 ^c^	4862.52 ± 6.29 ^b^	4682.13 ± 26.02 ^d^	4539.23 ± 1.44 ^f^	4922.49 ± 9.66 ^a^	4448.82 ± 28.99 ^g^

Umami amino acids: Glu and Asp; sweet amino acids: Ser, Gly, Thr, Ala; bittersweet amino acids: Arg, Pro, Met, Val, Lys; bitter amino acids: His, Tyr, Ile, Leu, Phe, Trp [46]. Different lowercase letters in the rows indicate significant differences (*p* < 0.05) among different cooking fish sauces.

## Data Availability

The original contributions presented in this study are included in the article. Further inquiries can be directed to the corresponding authors.

## References

[1] Li C., Li W., Li L., Chen S., Wu Y., Qi B. (2022). Microbial community changes induced by a newly isolated salt-tolerant *Tetragenococcus muriaticus* improve the volatile flavor formation in low-salt fish sauce. Food Res. Int..

[2] Ma X., Zhang Y., Li X., Bi J., Zhang G., Hao H., Hou H. (2022). Impacts of salt-tolerant *Staphylococcus nepalensis* 5-5 on bacterial composition and biogenic amines accumulation in fish sauce fermentation. Int. J. Food Microbiol..

[3] Wang X., Meng Q., Song H. (2022). Characterization of odor-active compounds in high-salt liquid-state soy sauce after cooking. Food Chem..

[4] Meng Q., Zhou J., Gao D., Xu E., Guo M., Liu D. (2022). Desorption of nutrients and flavor compounds formation during the cooking of bone soup. Food Control.

[5] Yang F., Chen E., Fu A., Liu Y., Bi S. (2025). Formation of key aroma compounds in *Agrocybe aegerita* during hot air drying: Amino acids and reducing sugars identified as flavor precursors. Food Chem..

[6] Han J., Kong T., Wang Q., Jiang J., Zhou Q., Li P., Zhu B., Gu Q. (2022). Regulation of microbial metabolism on the formation of characteristic flavor and quality formation in the traditional fish sauce during fermentation: A review. Crit. Rev. Food Sci. Nutr..

[7] Lu C., Zhang Y., Zhan P., Wang P., Tian H. (2023). Characterization of the key aroma compounds in four varieties of pomegranate juice by gas chromatography-mass spectrometry, gas chromatography-olfactometry, odor activity value, aroma recombination, and omission tests. Food Sci. Hum. Wellness.

[8] Geng Q., Qin F., Zeng M., Wang Z., Chen Q., Zhang W., Zhu B., He Z., Chen J. (2024). Effects of heat treatment on the profile of volatile flavor components in bamboo shoots. Food Biosci..

[9] Feng S., Li P., Li Y., Xiao X., Chen X., Leng H., Liang H., Zhou L., Chen T., Ding C. (2025). Volatile profiles and characteristic odorants in camellia seeds with different heat pretreatments. Food Chem..

[10] Xu L., Wu G., Huang J., Zhang H., Jin Q., Wang X. (2023). Sensory-directed flavor analysis of key odorants compounds devel-opment of French fries and oils in the break-in, optimum and degrading frying stage. Food Sci. Hum. Wellness.

[11] Zhang M., Chen M., Fang F., Fu C., Xing S., Qian C., Liu J., Kan J., Jin C. (2022). Effect of sous vide cooking treatment on the quality, structural properties and flavor profile of duck meat. Int. J. Gastron. Food Sci..

[12] Hu G., Chen J., Du G., Fang F. (2023). Moromi mash dysbiosis trigged by salt reduction is relevant to quality and aroma changes of soy sauce. Food Chem..

[13] Sissons J., Davila M., Du X. (2022). Sautéing and roasting effect on free amino acid profiles in portobello and shiitake mushrooms, and the effect of mushroom- and cooking-related volatile aroma compounds on meaty flavor enhancement. Int. J. Gastron. Food Sci..

[14] (2014). National Food Safety Standard Aquatic Seasoning.

[15] Wei Q., Liu G., Zhang C., Sun J., Zhang Y. (2022). Identification of characteristic volatile compounds and prediction of fermentation degree of pomelo wine using partial least squares regression. LWT.

[16] Andaleeb R., Zhang D., Jiang S., Zhang Y., Liu Y. (2023). Volatile profile and multivariant analysis of Sanhuang chicken breast in combination with Chinese 5-spice blend and garam masala. Food Sci. Hum. Wellness.

[17] Gong X., Mi R., Chen X., Zhu Q., Xiong S., Qi B., Wang S. (2023). Evaluation and selection of yeasts as potential aroma enhancers for the production of dry-cured ham. Food Sci. Hum. Wellness.

[18] Zhang X., Wang Z., Xu X., Mu X., Fu B., Xu J., Ye S., Du M. (2024). The relationships between flavor substances and microbial communities during the fermentation of Chinese traditional red sour soup. Food Biosci..

[19] Zhang Y., Yang Z., Zhao S., Gai J., Wang L., Ning X., Tao N. (2023). Quality characteristics of sardine (*Sardina pilchardus*) fish sauce produced using five kinds of material. Int. J. Gastron. Food Sci..

[20] Wu C., Hou H., Zhang Y., Yang Z., Wang L. (2025). Dynamics of bacterial composition and association with quality formation and histamine accumulation during *Antarctic krill* fish sauce fermentation. Int. J. Food Microbiol..

[21] Cui J., Wang Y., Wang Q., Yang L., Zhang Y., Karrar E., Zhang H., Jin Q., Wu G., Wang X. (2022). Prediction of flavor of Maillard reaction product of beef tallow residue based on artificial neural network. Food Chemistry: X.

[22] Wang X., Zhu L., Song X., Jing S., Zheng F., Huang M., Feng S., La L. (2023). Characterization of terpenoids and norisoprenoids from base and retail Qingke Baijiu by GC × GC-TOFMS and multivariate statistical analysis. Food Sci. Hum. Wellness.

[23] Li Y., Yuan L., Liu H., Liu H., Zhou Y., Li M., Gao R. (2023). Analysis of the changes of volatile flavor compounds in a traditional Chinese shrimp paste during fermentation based on electronic nose, SPME-GC-MS and HS-GC-IMS. Food Sci. Hum. Wellness.

[24] Kirkwood A., Fisk I., Xu Y., Reid J., Yang N. (2025). Mechanisms of aroma compound formation during the drying of *Dendrobium nobile* stems (Shihu). Food Chem..

[25] Li Y., Cao Z., Yu Z., Zhu Y., Zhao K. (2023). Effect of inoculating mixed starter cultures of *Lactobacillus* and *Staphylococcus* on bacterial communities and volatile flavor in fermented sausages. Food Sci. Hum. Wellness.

[26] Zhang D., Ayed C., Fisk I.D., Liu Y. (2023). Effect of cooking processes on tilapia aroma and potential umami perception. Food Sci. Hum. Wellness.

[27] Yu S., Song J., Hu T., Wang J., Liu X., Zheng Y., Shi L., Wan S., Wang M. (2022). Unraveling the core functional bacteria and their succession throughout three fermentation stages of broad bean paste with chili. Food Sci. Hum. Wellness.

[28] Li Y., Jiang S., Zhu Y., Shi W., Zhang Y., Liu Y. (2023). Effect of different drying methods on the taste and volatile compounds, sensory characteristics of *Takifugu obscurus*. Food Sci. Hum. Wellness.

[29] Hu H., Wang Y., Shen M., Huang Y., Li C., Nie S., Xie M. (2022). Effects of baking factors and recipes on the quality of butter cookies and the formation of advanced glycation end products (AGEs) and 5-hydroxymethylfurfural (HMF). Curr. Res. Food Sci.

[30] Deng S., Cui H., Hussain S., Hayat K., Liu W., Zhang X., Ho C.T. (2025). Promoted formation of pyrazines by targeted precursor addition to improve aroma of thermally processed methionine-glucose Amadori compound. Food Chem..

[31] Xing H., Yaylayan V. (2023). Mechanochemistry of Strecker degradation: Interaction of glyoxal with amino acids. Food Chem..

[32] Yang M., Chen X., Ding L., Wang F., Yu G. (2025). Mechanism and biodegradability of melanoidins formed in hydrothermal process: From model compounds to food waste. Bioresour. Technol..

[33] Gao R., Xue J., Shi T., Li Y., Yuan L. (2024). Effects of ‘bask in sunlight and dewed at night’ on the formation of fermented flavor in shrimp paste after maturation. Food Chem..

[34] Lu M., Sheng C., Ke H., Li T., Liu Q., Zhang J., Li L., Wang Y., Ning J. (2024). Revealing the differences in aroma of black tea under different drying methods based on GC–MS, GC-O. Food Chem. X.

[35] Huang W., Liu Q., Fu X., Wu Y., Qi Z., Lu G., Ning J. (2024). Fatty acid degradation driven by heat during ripening contributes to the formation of the “Keemun aroma”. Food Chem..

[36] Zhu S., Zhu L., Ke Z., Chen H., Zheng Y., Yang P., Xiang X., Zhou X., Jin Y., Deng S. (2023). A comparative study on the taste quality of *Mytilus coruscus* under different shucking treatments. Food Chem..

[37] Zhang D., Yang N., Fisk I.D., Li J., Liu Y., Wang W. (2022). Impact of cooking on the sensory perception and volatile compounds of *Takifugu rubripes*. Food Chem..

[38] Chew S.C., How Y.H., Chang L.S., Tan C.H., Chuo K.M.J., Wong S.Y.W., Degraeve P., Nyam K. (2024). The impact of cooking methods on the physical, sensory, and nutritional quality of fish. Int. J. Gastron. Food Sci..

[39] Guo X., Ho C.T., Wan X., Zhu H., Liu Q., Wen Z. (2021). Changes of volatile compounds and odor profiles in Wuyi rock tea during processing. Food Chem..

[40] Li J., Zhang Q., Peng B., Hu M., Zhong B., Yu C.W., Tu Z. (2023). Exploration on the quality changes and flavour characteristics of freshwater crayfish (*Procambarus clarkia*) during steaming and boiling. LWT.

[41] Wang H., Yu C., Sun Y., Cui N., Zhong B., Peng B., Hu M., Li J., Tu Z. (2024). Characterization of key off-odor compounds in grass carp cube formed during room temperature storage by molecular sensory science approach. Food Chemistry: X.

[42] Zhang Q., Gu H., Mu H., Zhao M., Chen Y., Gao X. (2022). Analysis of volatile flavor components of traditional Chinese fish sauce during fermentation by GC-MS. China Brewing.

[43] Ma S., Ding C., Zhou C., Shi H., Bi Y., Zhang H., Xu X. (2024). Peanut oils from roasting operations: An overview of production technologies, flavor compounds, formation mechanisms, and affecting factors. Heliyon.

[44] Muthee M.W., Khamis F.M., Cheseto X., Tanga C.M., Subramanian S., Egonyu J.P. (2024). Effect of cooking methods on nutritional value and microbial safety of edible rhinoceros beetle grubs (*Oryctes* sp.). Heliyon.

[45] Niu F., Lin C., Liao H., Zhang B., Zhang J., Pan W. (2025). Formation mechanism of giant squid myofibrillar protein aggregates induced by egg white protein during heat treatment. Food Hydrocoll..

[46] López-Martínez M.I., Toldrá F., Mora L. (2025). Comparative analysis of different pretreatments and hydrolysis conditions for the generation of taste-related substances in pork liver hydrolyzates. Food Chem..

[47] Zhou Y., Xu Y., Xia W., Yu D., Wang B., Xu J. (2024). Insight into the role of lipids in odor changes of frozen grass carp (*Ctenopharyngodon idella*) based on lipidomics and GC–MS analysis: Impact of freeze-thaw cycles and heat treatment. Food Chem..

